# Zebrafish Unga Is Required for Genomic Maintenance upon Genotoxic Stress and Male Fertility

**DOI:** 10.3390/jdb13030032

**Published:** 2025-09-02

**Authors:** Latifa Kazzazy, Flóra Huba, Bálint Lóránt Hausz, Dávid Mező, Viktória Perey-Simon, Bálint Jezsó, Abdulrahman Seddik, Zoran Marinović, Judit Tóth, Angéla Békési, Beáta G. Vértessy, Máté Varga

**Affiliations:** 1 Department of Genetics, ELTE Eötvös Loránd University, 1117 Budapest, Hungary; latifakazzazi@gmail.com (L.K.); huba.flora@gmail.com (F.H.); abdulrahman.m.alsadiq@gmail.com (A.S.); 2 Department of Veterinary Medical Sciences, University of Bologna, 40126 Bologna, Italy; balintlorant.hausz2@unibo.it; 3 Institute of Molecular Life Sciences, HUN-REN Research Centre for Natural Sciences, 1117 Budapest, Hungary; perey-simon.viktoria@ttk.hu (V.P.-S.); toth.judit@ttk.hu (J.T.); bekesi.angela@vbk.bme.hu (A.B.); vertessy.beata@ttk.hu (B.G.V.); 4 Department of Applied Biotechnology and Food Science, Faculty of Chemical Technology and Biotechnology, Budapest University of Technology and Economics, 1111 Budapest, Hungary; 5 Department of Biochemistry, ELTE Eötvös Loránd University, 1117 Budapest, Hungary; jezso.balint@ttk.elte.hu; 6 Department of Zoology, Faculty of Science, South Valley University, 83523 Qena, Egypt; 7 Department of Aquaculture, Institute of Aquaculture and Environmental Safety, Hungarian University of Agriculture and Life Sciences, 2100 Gödöllő, Hungary; marinovic.zoran@uni-mate.hu

**Keywords:** zebrafish, base excision repair, BER, UNG, genome maintenance, fertility

## Abstract

DNA repair is a multifaceted biological process that involves multiple pathways to counter the types of damage the genome encounters throughout life. In the past decade zebrafish became a popular model organism to study various aspects of vertebrate DNA repair, and the characterization of several mutant lines deficient in key players of the repair pathways has significantly contributed to our understanding of the roles the corresponding proteins play in the maintenance of genomic integrity. Interestingly, the base-excision repair (BER) pathway remained one of the less characterized DNA repair processes in fish. Here we provide a detailed characterization of zebrafish deficient in one of the key components of BER, the uracil-DNA glycosylase Unga. We show that while these fish are viable, they display an altered response to genotoxic stress and *unga* mutant males show an interesting form of subfertility.

## 1. Introduction

The genomes of living beings are continuously exposed to external and internal mutagenic processes, such as oxidation, deamination, alkylation, and error-prone replication; therefore, DNA repair is crucial for preserving the integrity of the genome. Base excision repair (BER), nucleotide excision repair (NER), mismatch repair (MMR), translesion synthesis (TLS), homologous recombination (HR), and non-homologous end-joining (NHEJ) are some of the main DNA repair pathways that eukaryotes use to shield their genomes from the consequences of those mutagenic processes [1].

Zebrafish (*Danio rerio*) have emerged as a useful vertebrate model system to study numerous developmental and cellular processes, including the aforementioned repair mechanisms [2,3,4]. The physiological traits that make this species an ideal model organism (i.e., short generation time, fecundity, external fertilization, transparency), coupled with a quickly expanding genetic toolkit, have made it essential for preclinical research [5,6,7,8,9,10]. The relatively high similarity between zebrafish and human genomes (~71% of protein coding genes and 82% of disease coding genes have at least one zebrafish ortholog), also helped this teleost species to become a popular model for the study of human diseases, including those linked to the dysfunction of DNA repair [11,12,13,14] (Of note, however, is that due to a recent teleost-specific genome duplication, some disease-linked genes have two zebrafish paralogs, making the modeling of such diseases more difficult).

Over the past decade, zebrafish mutants of DNA repair genes have also provided important insights into the developmental roles of these genes and their involvement in the response to DNA damage. Interestingly, in zebrafish, genes primarily involved in genome maintenance often also impact sex determination (SD) and differentiation (SDiff). For example, knockout of the genes associated with the Fanconi anemia (FA) pathway, important for the resolution of interstrand crosslinks, leads to complete or partial male bias, and masculinization can also be observed in homozygous mutants for *rad51*, which encodes a protein essential for strand invasion and crossing over [15,16,17,18,19]. Our group has also recently demonstrated that loss-of-function mutations affecting *blm*, the zebrafish ortholog encoding the RecQ-type helicase Blm, affect lifespan, and *blm* is also essential during the exponential proliferation of germ cells (GCs) in juveniles and during meiosis in males [20].

A recent high-throughput study on the phenotypic characterization of zebrafish mutants of DNA repair genes, has also identified six genes (*blm*, *brca2*, *fanci*, *rad51*, *rad54l*, and *rtel1*) that are crucial for proper SD, three genes (*atad5a*, *ddb1*, and *pcna*) that are necessary for proper embryonic development and hematopoiesis, and further seven genes (*apex1*, *atrip*, *ino80*, *mre11a*, *shfm1*, *telo2*, and *wrn*) that are important for growth and development during the juvenile stage. Hypersensitivity to DNA-damaging agents was also demonstrated in the mutants of six DNA repair genes (*atad5a*, *brca2*, *polk*, *rad51*, *shfm1*, and *xrcc1*) [21].

Finally, the loss of MMR function in zebrafish mimics some characteristics of human diseases linked to MMR deficiencies, such as hereditary nonpolyposis colorectal cancer and neurofibromatosis syndrome. Zebrafish with mutations in MMR genes, such as *mlh1*, *msh2*, and *msh6*, develop a variety of tumors, primarily neurofibromas and malignant peripheral nerve sheath tumors [22].

The BER pathway is chiefly accountable for repairing DNA base damage resulting from oxidative and alkylating stress, and importantly, spontaneous base deamination. Cytosine deamination is a common spontaneous DNA lesion where cytosine (C) is converted to uracil (U). This process contributes significantly to mutagenesis and genome instability. The background rate of cytosine deamination under physiological conditions yields ~12 mutagenic uracils per day per cell in a human diploid cell in a double-stranded DNA context [23]. However, the rate of deamination is significantly faster in an oxidative environment and is measured to be 140-fold faster in single-stranded DNA [23]. These spontaneous deamination events are a significant source of C→T transition mutations and have profound implications for genome stability and evolution. Such erroneous nucleobases are recognized by uracil-DNA glycosylases (UDGs), which will extricate the respective base from the double helix, and hydrolyze the glycosidic bond linking the base to the deoxyribose. Four unique UDGs have been identified in mammalian cells (UNG, SMUG1, TDG, and MBD4), which act depending on the specific cellular and molecular context [24,25].

UNG is the most efficient UDG for removing uracil from both single- and double-stranded DNA. UNG has an elevated expression during the S-phase and is reported to interact with replication protein A (RPA2) and proliferation cell nuclear anti-gene (PCNA), which allows for the efficient post-replicative removal of accidentally incorporated uracils [26,27]. Its order of substrate preference and high specificity for U suggest that its primary biological role is in the repair of U arising from either cytosine deamination or thymine replacing misincorporation [28]. In contrast, other UDGs, having lower activities and altered substrate preferences, are more specialized for their roles. SMUG1 and TDG are less selective for uracil and can also hydrolyze other derivatives like hydroxymethyl-uracil [28]. TDG and MBD4 have strong preferences for U(X):G mismatches [29]. UNG, TDG, and MBD4 have been implicated in DNA demethylation [30,31], while MBD4 has a methyl CpG binding domain that narrows the genomic targets of this highly specialized UDG [31]. The zebrafish orthologue of UNG is uracil-DNA glycosylase A (Unga), while another UNG-like paralogue, the Ungb, is more diverged, less expressed, and less characterized. In zebrafish, Unga is maternally deposited and shows strong expression from the one-cell stage through gastrulation [32,33].

Excision by UDGs results in an apyrimidinic/apurinic (AP) site, subsequently processed by AP endonucleases (APE1 and APE2). These nucleases cleave the sugar-phosphate backbone 5′ of the abasic site, resulting in a nick close to the lesion [24]. Failing to initiate the repair process may pose significant risks to the cell, as AP sites can induce polymerase stalling, necessitating mutagenic translesion DNA synthesis for bypass, while not efficiently resolved repair intermediates may result in double-strand breaks [34,35,36].

The repair itself may be conducted using one of the following methods: short-patch or long-patch BER. In short-patch BER, DNA polymerase β (Polβ) integrates the correct nucleotide into the abasic site and removes the remaining deoxyribose phosphate (dRP) via its lyase activity. Ultimately, DNA ligase III (Lig3) and its co-factor XRCC1 finalize the repair by sealing the DNA backbone [37]. In the case of long-patch BER, DNA polymerases δ, β, or ε, in conjunction with PCNA, synthesize an elongated DNA segment from the apurinic/apyrimidinic (AP) site, displacing the downstream nucleotide sequence, including the 5′ deoxyribose phosphate (dRP) residue from the base excision, creating a flap. The flap is then excised by flap endonuclease 1 (Fen1), and unlike short-patch BER, DNA ligase 1 (Lig1) is enlisted to ligate the backbone [24,36]. The selected polymerase depends on several factors, including its availability and post-translational modifications [36].

The analysis of BER pathway zebrafish mutants has received less attention. An initial, comprehensive analysis of BER in early zebrafish development has shown that the process is more efficient in adults than in eggs, and replicative polymerases dominate BER after fertilization. Polβ is absent from early embryos and appears only at the mid-blastula transition, after the onset of zygotic transition [38]. At the same time, UDGs are active in the early embryo and can remove genomic uracil. The only existing report of a functional knockdown of Unga in zebrafish using anti-sense synthetic morpholino oligonucleotides suggested that Unga loss-of-function affected DNA demethylation in embryos and interfered with the dynamics of zygotic transcription [33]. This report also provided evidence about the functionality of Unga as a bona fide UDG and demonstrated that demethylation was dependent on glycosylase activity. Recently, however, morpholino-based knockdown experiments in zebrafish have been shown to produce off-target and nonspecific artifacts that complicate result interpretation [39,40]. Consequently, current standards of the field now require morpholino-based studies to be validated with authentic mutant lines. Until now, such a mutant line has been unavailable for *unga*.

In this study, we demonstrate that, in contrast to previously reported *unga* morphants [33], homozygous *unga* knockout zebrafish develop normally, but Unga-deficiency makes them more sensitive to environmental mutagens, and *unga* mutant males show reduced fertility.

## 2. Materials and Methods

### 2.1. Fish Husbandry and Maintenance

Wild-type (AB) and *unga^elu24^* mutant zebrafish strains used in this study were maintained and bred in the animal facility of the Biology Institute of ELTE Eötvös Loránd University, adhering to standards of the field [41,42]. Experiments were carried out in accordance with the Hungarian Act of Animal Care and Experimentation (1998, XXVIII) and with directive 2010/63/EU of the European Parliament and of the Council of 22 September 2010 on the protection of animals used for scientific purposes. All animal husbandry protocols used for this study were approved by the ELTE Animal Welfare Animal Committee and the Hungarian National Food Chain Safety Office (PEI/001/1713-2/2015).

### 2.2. Size Measurements

Size measurements of wild-type and *unga* mutant zebrafish were performed using a precision caliper to guarantee reliable data collection. The fish were submerged in a 120 mg/L tricaine methane-sulfonate (MS-222) anesthetic solution (Merck KGaA, Darmstadt, Germany, E10521). After achieving a deep anesthetic state, length measurements were recorded with the fish positioned on its right side, snout to the left on a measuring board. The fish were straightened, and the measurements were taken with the caliper from the snout to the base of the caudal fin (standard length).

### 2.3. Genome Editing and Genotyping

We used CRISPR/Cas9-based mutagenesis to target the zebrafish *unga* gene as described before [43]. Custom Alt-R crRNAs and standard tracrRNA were purchased from Integrated DNA Technologies (IDT). Cas9 RNPs were assembled by mixing 1 µL of Cas9 protein (10 μM, New England Biolabs, Ipswich, MA, USA, M0646T), 0.7 µL of tracRNA:crRNA hybrid (57 μM), and 1.3 µL of ddH_2_O to achieve a final volume of 3 µL. Two nanoliters of the RNP mixture was injected per embryo at the 1–2 cell stage. The targeted sequence (with PAM sequence in bold): 5′-GCTCCAACTGCTCAGGACTCAGGG-3′.

The forward and reverse primers used to test the efficiency of CRISPR-targeting and later to genotype carriers and homozygotes, were as follows: (Fwd) 5′-GCACAAATGCCACGAATAGTTT-3′ and (Rev) 5′-AGCTCTTCCCGAATTCAGCACT-3′. For PCR-based genotyping the following wild-type specific and mutant-specific forward primers were combined with the aforementioned reverse primer: (Wt-Fwd) 5′-AATGAGGCACCCCTGAGTCCTG-3′ and (Mut-Fwd) 5′-GAAATGAGGCACCCCTGAGCAG-3′. Genomic DNA templates were extracted from ~10 embryos at the 2 dpf stage (to confirm targeting efficiency) or from caudal tail lysates (for genotyping) as described before [44].

### 2.4. Reverse Transcription and Quantitative PCR (qPCR)

For the quantitative analysis of *unga* transcription we used a reverse transcribed cDNA library of zebrafish transcripts from 24 h post fertilization (hpf) embryos as a template. Total RNA was isolated from 100 wild-type or *unga^elu24/elu24^* mutant embryos using TRIzol reagent (Zymo Research, Irvine, CA, USA, 03312023), following the manufacturer’s protocol. Reverse transcription was performed using a Maxima H Minus Reverse Transcriptase Kit (Thermo Fisher Scientific, Waltham, MA, USA, EP0751), starting with 1 μg total RNA and using oligo(dT) primers. The qPCR reaction was assembled in a Maxima SYBR Green qPCR Master Mix (Thermo Fisher Scientific, Waltham, MA, USA, K0223) and run in a Roche LightCycler 96 machine. The primers used to amplify the *unga* transcripts were as follows: 5′- CTGTGGTGCACTGGCTCAGCTC-3′ and 5′- CGGTGAGCAGACAGTGGTGACG-3′. As a reference, we used the *lsm12b* gene, which has been validated before for the analysis of gene expression during zebrafish development [45]. Three independent biological samples were used for both control and mutant conditions.

### 2.5. Bioinformatic Analysis

For BLAST comparison of zebrafish *unga*, *ungb*, and human *UNG* coding sequences (CDSs) and the Unga, Ungb, and UNG proteins, the corresponding sequences from the Ensembl database were used (ENSDART00000062359.5 and ENSDART00000147850.3 from GRCz11—v114.11, and ENST00000242576.7 from GRCh38.p14—v114.38). Phylogenetic analysis of the *UNG* orthologs was performed using the inbuilt Ensembl “Gene gain/loss tree” option, see: https://may2025.archive.ensembl.org/Danio_rerio/Gene/SpeciesTree?db=core;g=ENSDARG00000042527;r=5:67351219-67365750 (accessed on 25 August 2025).

Cap analysis of gene expression (CAGE-Seq) datasets were visualized using the inbuilt Genome Data Viewer (GDV) of the GRCz12tu dataset hosted on the National Center for Biotechnology Information (NCBI) website of the National Institute of Health (NIH): https://www.ncbi.nlm.nih.gov/datasets/genome/GCF_049306965.1/. Datasets are available through the GDV interface and can be accessed directly: for *unga* (https://short-link.me/18q2l) and for *ungb* (https://short-link.me/18q31) (links accessed on 25 August 2025).

To assay maternal and zygotic transcripts, we reanalyzed a previously published SLAM-Seq [thiol(SH)–linked alkylation for the metabolic sequencing of RNA] dataset [46] using R-Studio [47].

Single-cell sequencing data from the Daniocell [48] dataset was reanalyzed using the *Seurat* package [49], and the related gene-ontology (GO) analysis was performed with *clusterProfiler* [50]. We also reanalyzed already published single-cell datasets for zebrafish ovaries and testes using the interactive applications provided in the respective publications [51,52]. To test for the expression of *unga* orthologs in other teleost species we relied on the PhyloFish database [53]. The corresponding phylogenetic tree was edited using the interactive Tree of Life website [54].

Data visualization were performed using *ggplot2* [55] and *ggpubr* [56] in RStudio (version 2024.12.0+467; Posit Software, Boston, MA, USA) and the figures were assembled in Affinity Designer (version 1.10.4; Pantone LLC, Carlstadt, NJ, USA).

### 2.6. Diepoxybutane (DEB) Treatments and Acridin Orange Stainings

DEB (Merck KGaA, Darmstadt, Germany, 202533), a well-characterized mutagenic reagent [16] was applied at a 1.5 mg/mL concentration to the medium of fish embryos at 6 hpf. The next day (18 h later) we added 1-phenyl 2-thiourea (PTU) to the medium to block pigmentation. At 2 dpf, the embryos were stained with Acridine Orange (Merck KGaA, Darmstadt, Germany, MKBS4724V) for half an hour. For adult testes, two wild-type control and two *unga* mutant adult males were randomly selected and euthanized. Their testes were dissected, placed in PBS, and immediately stained with AO for 20 min. After washing, each testis was placed on a slide and imaged. All specimens were photographed under a Zeiss Stereo Lumar microscope at 488 nm wavelength.

Image processing was performed in Fiji [57], and the results were used to calculate the optimal threshold for foci detection.

### 2.7. Histology

For histological examination, 2 dpf embryos, or testes dissected from adult males, were fixed in phosphate-buffered saline (PBS) containing 4% paraformaldehyde. Fixed samples were embedded using JB-4 resin (Merck KGaA, Darmstadt, Germany, EM0100) according to the manufacturer’s protocol. Sections 10 μm thin were cut using a Leica rotation microtome. Embryo samples were stained with hematoxylin and eosin (HE), while the testis sections were stained with Toluidine Blue [58].

Sections were photographed using a Nikon Eclipse 80i microscope and SPOT RTKE 7.4 Slider camera.

### 2.8. Sperm Motility Measurements

For the spermatozoa concentration and motility measurements five-five wild-type and *unga^elu24/elu24^* homozygous mutant male donors were randomly selected. The fish were placed in an anesthetic water bath with a terminal concentration (400 mg/L) of tricaine methane-sulfonate (MS-222—Merck-Sigma, E10521) [59,60]. Upon reaching the deep anesthetic plane, observed by equilibrium loss, loss of response to stimuli and slowed gill movements, the fish were fixed in a humid sponge under an optical stereo microscope (SMZ745, Nikon Corporation, Tokyo, Japan) with their abdomen facing upwards, rinsed with system water to avoid contamination with the anesthetic solution, and dried with a paper towel to avoid the activation of the seminal sample. For the collection, borosilicate glass capillary tubes (World Precision Instruments, Sarasota, FL, USA, Kwik-Fil^TM^) were pre-marked at the capillary volume equal to 1 µL content and fixed hermetically in a Pasteur pipette (Brand GmbH + CO KG, Wertheim, Germany). The capillary tube was placed vertically onto the urogenital area and careful suction was applied with the Pasteur pipette while the fish was gently massaged from the abdomen towards the urogenital area with an unhinged titanium tweezer (Vve Dumont Fs & Cie Succ; Dumont SA, Montignez, Switzerland) previously sterilized in 70% ethanol. The obtained seminal sample was immediately diluted to 1:10 in microtubes with cyprinid immobilizing solution (200 mM KCl, 30 mM, pH 8) [61,62] that was chilled to 4 °C prior of usage [63,64].

The spermatozoa motility measurement was performed by CASA (computer assisted sperm analysis) using a 100× magnification negative Phase contrast objective (BA310, MoticEurope S.L.U; Barcelona, Spain), an avA1000-100gc camera (Basler, Ahrensburg, Germany), and the AndroVision^®^ software (reference: 12500/0000, Minitüb GmbH, Tiefenbach, Germany). The activating media consisted of system water mixed with Bovine Serum Albumin (1 mg/mL; Capricorn Scientific GmbH, Ebsdorfergrund, Germany). Nine µL of activating media was loaded into the Makler^®^ counting chamber (Microptic S.L.U., Barcelona, Spain). Then, 1 µL of the diluted sample was loaded and mixed with the activating media. The analysis was performed in the first 30 s upon activation, until at least 500 spermatozoa were captured. The software operated with the following settings: (1) size of head area: 1–100 µm; (2) 1 pixel = 0.6622 µm; and (3) 0.5 s capture time. Spermatozoa were considered immotile if curvilinear velocity (VCL) was less than 1 µm/second or curvilinear distance (DCL) was less than 1 µm. Spermatozoa were considered progressively motile if VCL was less than 13 µm/s.

For the spermatozoa concentration measurements, the diluted sample was further diluted to 1:100 with the immobilizing solution and mixed well with pipetting; then, 10 µL was loaded into bright-line improved Neubauer (Paul Marienfeld GmbH & Co. KG, Lauda-Königshofen, Germany) chambers in duplicate. The evaluation was performed by two different persons under a 400× magnification light microscope objective (Leica DMRB, Leica Microsystems GmbH, Wetzlar, Germany). After averaging the counted sperm cells from four big squares of the Neubauer chamber, the original sperm concentration was obtained by multiplying the average number of cells with the dilution factor (×1000) and with the conversion ratio from the chamber volume to ml (×10^4^). The two independent evaluations of the same ejaculate were averaged to minimize personal bias and reported as spz/mL.

## 3. Results

### 3.1. UNG Paralogs in the Zebrafish Genome

The most recent Ensembl build of the zebrafish genome (GRCz11, v114.11) and the recently released GRCz12tu NCBI RefSeq telomere-to-telomere assembly (GCF_049306965.1) both contain two annotated *UNG* paralogs on chromosome 5. Of these two, the *unga* gene is in a more telomeric position [chr5(-): 73,362,689–73,377,220, GRCz12tu] than *ungb* [chr5(+): 32,515,610–32,567,165, GRCz12tu].

The presence of the two *UNG* paralogs in the zebrafish genome would be compatible with the teleost-specific whole genome duplication event (3R) in the early-middle Permian, which was followed by a slow and partial re-diploidization of the genome [65,66]. However, a closer analysis suggests that not all teleost genomes show signs of a 3R-related *UNG* duplication, and the two paralogs in the zebrafish genome are the result of a later gene duplication event affecting only the Otocephala clade (Figure S1).

A sequence comparison of the two predicted polypeptides revealed that the Ungb protein also appeared to be missing 42 amino acids in its N-terminal and the rest of the two sequences were 65.6% identical (164/250) (The BLAST score for comparing the two CDSS is 195). For comparison, the human UNG and Unga show a higher similarity (67%-209/313) over the entire length of the two proteins (The total BLAST score for the respective CDSs is 498). As the 44 N-terminal residues are important for the nuclear localization of UNG [67], we can conclude that even if expressed, Ungb would be cytoplasmic and therefore unable to perform its DNA-glycosylase activity.

**Figure 1:**
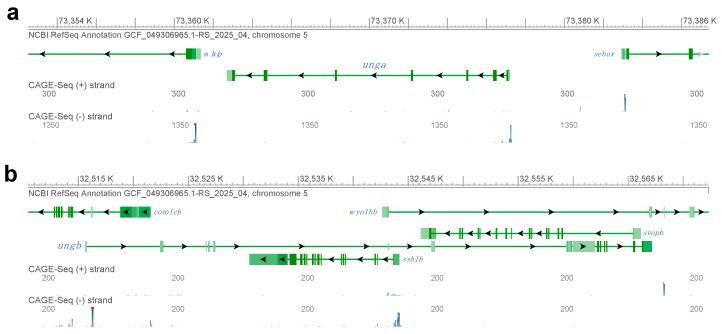
Genomic analysis of zebrafish *UNG* orthologs. (**a**) The genomic region encoding *unga* on chromosome 5. CAGE-Seq data clearly indicate active transcription from the annotated transcriptional start site of the gene. (**b**) A detailed look at the genomic region with the annotated *ungb* gene reveals multiple other genes that overlap the annotation. We also note that while many of these other genes are actively transcribed, as suggested by the mapping of CAGE-Seq datasets, an active transcriptional start site is not visible for *ungb*. (Figure was generated with GDV, for links and details see Materials and Methods section).

To understand if either of the paralogs is transcribed, we also took advantage of the CAGE-Seq data available through the NCBI Genome Data Viewer (Figure 1). Interestingly, while for *unga* and its neighbors we could identify clear peaks at the annotated transcriptional start sites (Figure 1a), in the case of *ungb* no such signal could be seen (Figure 1b). We also note that the *ungb* annotation overlaps with several other genes, mostly transcribed in an anti-sense orientation (e.g., *coro1cb*, *ssh1b* and *svopb*), some of which show signs of transcription themselves (e.g., *ssh1b*). Of note is that examining a wide variety of transcriptomic datasets (see below) also did not reveal significant transcription from the *ungb* locus at any developmental stage.

Altogether these observations suggest that while *unga* encodes a bona fide ortholog of *UNG*, the genomic sequence containing the annotated zebrafish *ungb* paralog is undergoing fast evolutionary changes, and *ungb* is most likely unfunctional in the genome of extant zebrafish and even if transcribed, its product cannot function in DNA Repair

### 3.2. Expression of Zebrafish UNG Orthologs During Development

**Figure 2:**
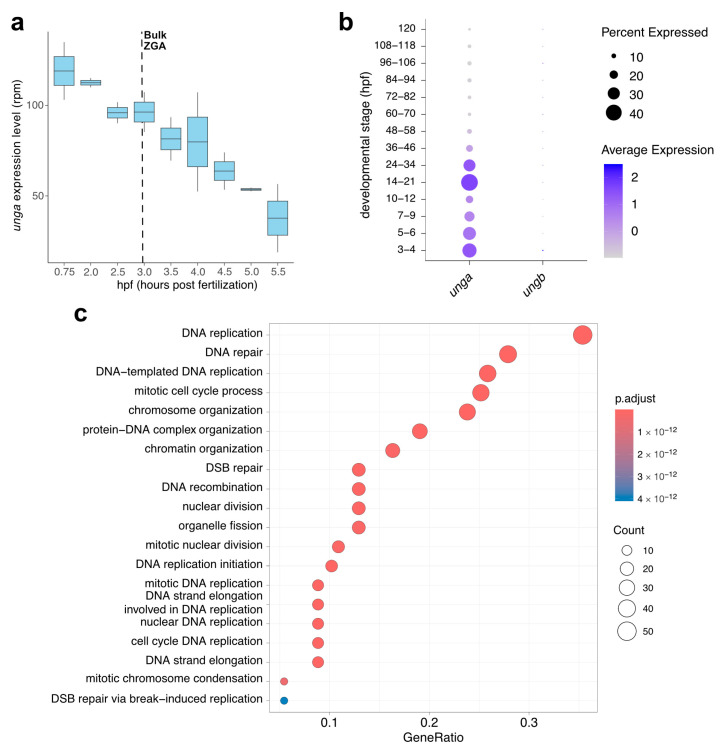
*In silico* analysis of the developmental expression of zebrafish *UNG* paralogs. (**a**) Expression dynamics of *unga* during the earliest stages of development show a gradual decrease in maternal transcript with no significant zygotic contribution after ZGA (Data from [46]). (**b**) The analysis of single-cell datasets also demonstrates the gradual clearance of maternal products, following a burst of *unga* expression during later stages of somatogenesis. During the same timeframe, *ungb* expression cannot be observed (Data from [48]). (**c**) GO analysis of genes that show significant co-expression with *unga* during early embryogenesis shows that the highest level of *unga* expression can be observed in actively dividing cell populations.

To reveal the developmental dynamics of *unga* and *ungb* expression, we have exploited some high-throughput datasets that became recently available. The in silico reanalysis of metabolically labeled mRNAs shows that *unga* is provided maternally (Figure 2a), while *ungb* expression is essentially non-detectable in current datasets [46,68,69]. Furthermore, while maternally provided *unga* levels gradually decline, zygotic transcription is not initiated during the zygotic genome activation (ZGA), and zygotic transcripts cannot be detected before the start of gastrulation.

This decline in the level of *unga* transcripts can also be validated by a close examination of the Daniocell database [48]. This single-cell RNA sequencing data suggests that *unga* levels decline until the middle of somitogenesis (12 hpf, corresponding to ~12 somite stage), and rebound afterwards (Figure 2b). The same dataset also demonstrates that essentially no *ungb* transcription can be observed during these stages of development.

The reanalysis of the Daniocell dataset also allowed us to test which genes were the most co-expressed with *unga* during the early stages of development. We performed a GO analysis on the genes that showed significant co-expression with *unga*, and these results demonstrated that the genes involved in processes related to cell division (e.g., DNA replication, nuclear division, chromosome organization) and DNA repair were significantly enriched in the cells that express *unga* (Figure 2c, Figurs S2 and Figure S3; Table S1). These results are also supported by earlier in situ hybridization data, which show *unga* expression in the proliferating regions of the nervous system, pharyngeal arches, fin buds, and the intestine [32,33].

### 3.3. Generation and Initial Characterization of a Novel Zebrafish Unga Allele

**Figure 3:**
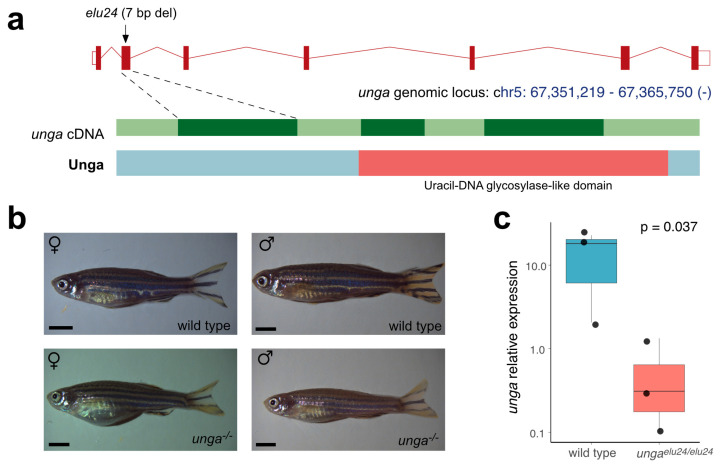
Creation and initial characterization of *unga* mutant zebrafish. (**a**) Targeted mutagenesis of *unga* results in a novel allele (*elu24*) carrying a 7 bp deletion in the second exon of the gene. (**b**) Wild-type and homozygous *unga^elu24/elu24^* adult zebrafish (scale bar: 0.33 cm). (**c**) The expression of *unga* is significantly reduced in *unga^elu24/elu24^* animals as shown by qPCR measurements (The Mann–Whitney test was used to calculate the *p*-values).

To characterize the role of *unga* in zebrafish development and homeostasis we created a deletion in the second exon of the gene using CRISPR/Cas9-based genome editing. The resulting allele (*elu24*) carries a 7 bp deletion (c.132_138delCCTGAGT), resulting in a frameshift and an early stop codon (Figure 3a and Figure S4).

After identifying the *elu24* allele in F0 fish, we created *unga^elu24/+^* carriers and crossed them to obtain homozygous mutant embryos. Interestingly, unlike in previously reported *unga* morphant animals [33], we did not observe delayed development and embryonic lethality. Homozygous *unga^elu24/elu24^* animals could be raised to adulthood, their gross morphology was normal, and we could identify both males and females amongst them (Figure 3b). Of note was that the adult body size of the mutant females was slightly smaller than the size of the age-matched controls. No size difference was observed in the case of the males (Figure S5).

Interestingly, however, the qRT-PCR analysis of the mutants confirmed an almost 25-fold reduction in the amount of *unga* transcripts (Figure 3c), suggesting that the frameshift and the resulting early stop codon trigger the nonsense-mediated decay (NMD) pathway in the mutant fish.

### 3.4. Zebrafish Lacking Functional Unga Allele Show Increased Sensitivity to Mutagens

**Figure 4:**
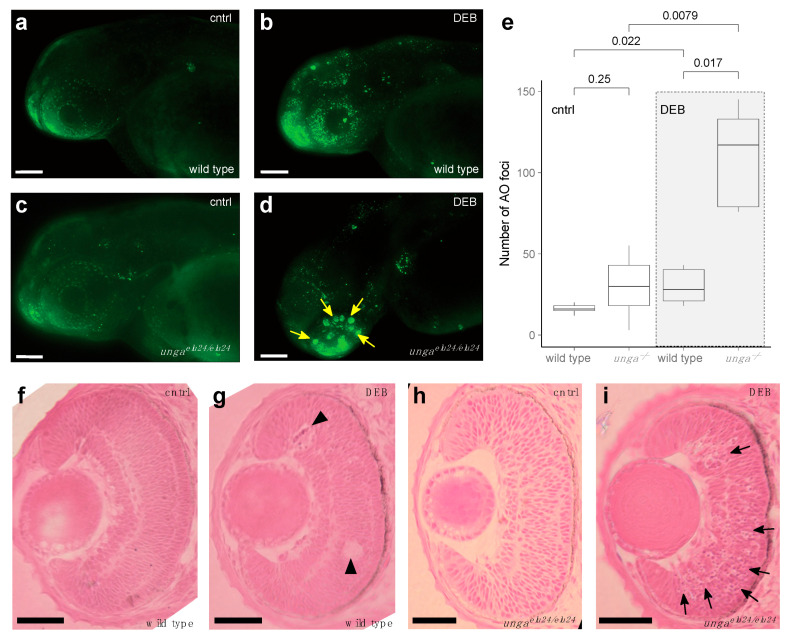
Sensitivity to DNA damage is increased in *unga* mutant zebrafish retinas. (**a**,**b**) AO staining of control and DEB-treated 2 dpf wild-type embryos shows an increased level of cell death upon the administration of the genotoxic agent. (**c**,**d**) DEB-treated *unga* mutant embryos also show elevated levels of apoptosis. However, unlike in wild-type controls, large clusters of AO-positive cells appear in these animals. Scale bar: 100 μm. (**e**) Quantification of apoptosis as measured by the number of AO foci in the control and DEB-treated embryos of the relevant genotypes (The Mann–Whitney test was used to calculate the *p*-values). (**f**–**i**) HE staining of the retinal sections of the wild-type and *unga* mutant embryos reveal that upon DEB treatment, a few dying (or missing) cells can be seen in the wild-type animals (black arrowheads). In contrast, large clusters of cells with apoptotic character are apparent in the mutant retinas (black arrows). Scale bar: 100 μm.

To test if Unga function was necessary for DNA repair in developing embryos, we first assayed for the number of dying cells in control and *unga*-deficient embryos using the nucleic acid-selective fluorescent dye Acridine Orange (AO). Our analysis showed that physiological programmed cell death levels in the homozygous *unga^elu24/elu24^* animals were comparably low to those observed in the wild-type controls (Figure 4a,c) (Of note was that as the homozygous mutant embryos were derived from the in-cross of *unga^elu24/elu24^* animals, in fact, these were maternal-zygotic mutants).

As our analysis did not reveal differences in the baseline levels of cell death (Figure 4a,c), we decided to trigger DNA damage by applying 1.25 μg/mL of the genotoxic agent diepoxybutane (DEB). Upon DEB treatment, we observed striking differences between the controls and *unga* homozygotes (Figure 4a–e). While cell death increased in all treated animals, large clusters of AO-positive cells were observed specifically in the retinas of the mutants (Figure 4b,d).

We performed a classical histological analysis on the retinas of 2 dpf control and DEB-treated, wild-type and *unga^elu24/elu24^* embryos, using HE staining. This revealed that in wild-type retinas, indeed, some dead or dying cells could be seen after the application of the genotoxic agent (Figure 4f,g), but in the mutant retinas, an abundance of shrinking cells (with apoptotic character) was apparent after DEB treatment. It is noteworthy that at this stage, retinal precursors are one of the fastest dividing cell populations within the developing embryo, which provides an important context to these observations.

In summary, our analysis shows that baseline levels of dying cells in 2 dpf *unga^elu24/elu24^* embryos are not elevated, but upon genotoxic treatment, a much more severe cell death phenotype will be apparent in the retinas of the mutants than in those of the controls.

### 3.5. Unga Impairment Results in Reduced Male Fertility in Zebrafish

**Figure 5:**
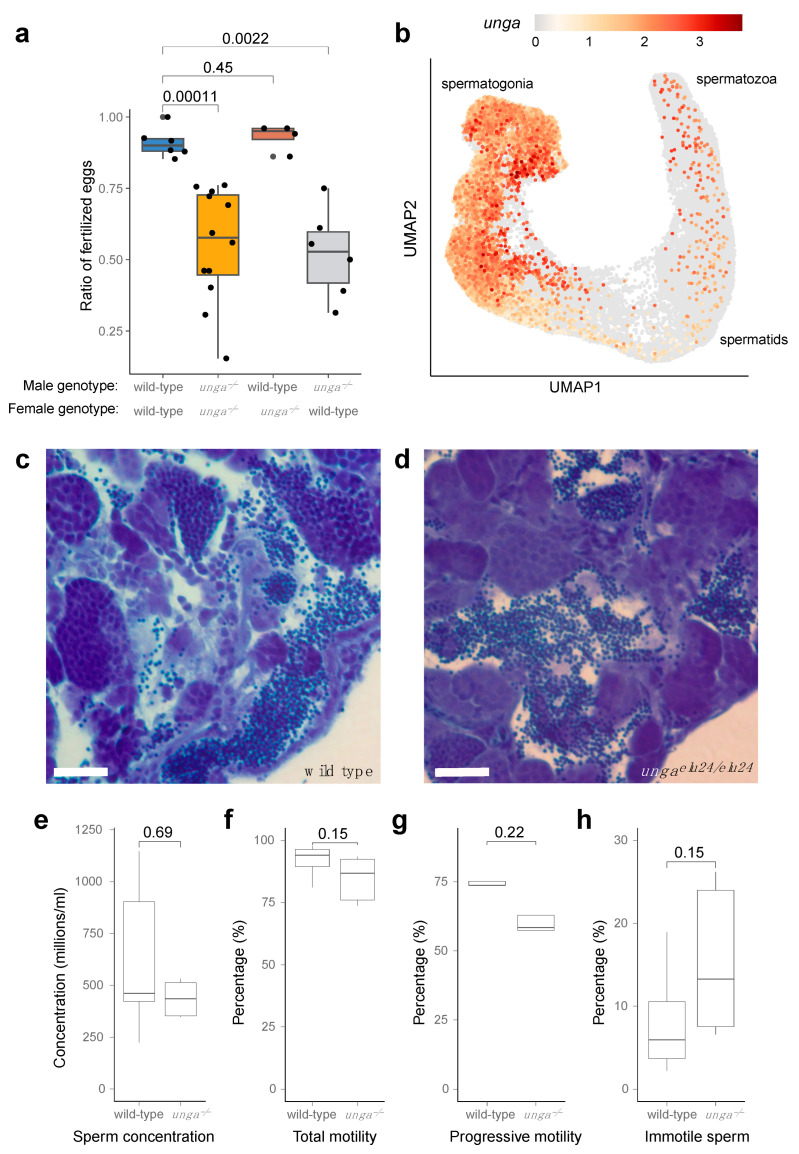
Homozygous *unga* mutant zebrafish males are subfertile. (**a**) Ratio of fertilized eggs in the indicated crosses. (**b**) Expression of *unga* in the differentiating germ cells of adult zebrafish. ScRNAseq data used in this reanalysis were derived from [52]. (**c**,**d**) Toluidine blue staining of testis sections from wild-type (**c**) and mutant (**d**) testes. Scale bar: 50 μm. (**e**–**h**) Comparative statistics of various sperm concentration (**e**) and motility (**f**–**h**) parameters between wild-type and *unga* mutant males (The Mann–Whitney test was used to calculate the *p*-values).

During our phenotyping efforts, we also observed subfertility when crossing the homozygous *unga^elu24/elu24^* adults, as often less than half of the embryos were fertilized (Figure 5a). As *unga* is expressed both in the female and male germ cells (Figure 5b and Figure S6), we decided to test mutant males and females separately, crossing them with wild-type fish. These crossings revealed that *unga^elu24/elu24^* females are not different in their fertility compared with the controls, whereas *unga^elu24/elu24^* male animals crossed with wild-type females show the same subfertility rate as observed in the in-crosses of the mutants.

The analysis of single-cell RNA sequencing datasets showed that both male and female germ cells expressed *unga* during their maturation (Figure 5b and Figure S6). In the testis, high expression levels are observed in spermatogonial cells. Although this expression gradually decreases during development, it does not completely disappear as the cells mature into differentiated spermatozoa (Figure 5b).

As an impairment of germ cell differentiation has been documented before in the testes of other zebrafish DNA-repair mutants [20], we also decided to check if spermatogonial differentiation was normal in the mutants. A histological analysis of the mutant testes showed that spermatozoa are present in them, despite the lack of Unga during the maturation process (Figure 5c,d), and no increase in cell death could be detected (Figure S7).

We also tested the overall concentration and motility of the sperm derived from the mutant males using automated, computer-assisted semen analysis (CASA), but none of tested parameters, such as concentration, (Figure 5e), total motility (Figure 5f), progressive motility (Figure 5g), or the ratio of immotile sperm (Figure 5h) showed a significant statistical difference.

## 4. Discussion

The efficient repair of DNA damage is essential for the genomic integrity of all living beings. Eukaryotes employ a variety of sophisticated DNA repair pathways to contend with various damage types, and understanding the mechanistic and genetic underpinnings of these pathways is essential for human health as well.

In the past couple of decades, the zebrafish has emerged as one of the top genetic model organisms that are used in preclinical studies aiming to understand monogenic and complex diseases. The high conservation of its genome maintenance mechanisms with those observed in other vertebrates, including us, humans, has also made it an ideal model to study defects in DNA repair pathways [2,3,4,15,16,17,18,19,20,21].

Interestingly, modeling BER-related conditions has received less attention, and while some notable studies have been performed [33,38], the characterization of bona fide zebrafish mutants in genes important for this essential pathway has been missing. UDGs play a pivotal role in maintaining genomic stability by initiating the BER pathway to remove uracil from DNA. Uracil can appear in DNA either via misincorporation during replication or through cytosine deamination [70]. In mammals, the enzymes UNG, SMUG1, TDG, and MBD4 exhibit overlapping substrate specificities but possess distinct affinity profiles. Consistent with this, studies suggest a finely tuned division of labor among them that varies according to the cellular context.

We provide here a thorough characterization of the two annotated zebrafish *UNG* orthologs, *unga* and *ungb*, providing evidence that only the former encodes a functional uracil-DNA glycosylase (Figure 1 and Figure 2) and also presenting the first characterization of a loss-of-function *unga* allele (Figure 3). We show that the lack of Unga does not directly affect the viability of embryos, and unlike many other genes involved in DNA repair, *unga* mutants do not show distorted sex ratios. Interestingly, however, we also note that the absence of Unga leads to the appearance of large apoptotic cell clusters in the retinas of embryos upon treatment with genotoxic agents, suggesting that overall levels of DNA repair are compromised in the mutants (Figure 4).

We also noticed subfertility in adult *unga* mutant males, but not females, suggesting an important role of Unga in the maturation of zebrafish spermatozoa (Figure 5). Unexpectedly, spermatogenesis per se is unaffected, as mutant males produce a normal number of spermatozoa. We observed only a slight, but statistically insignificant change in the motility of the mutant spermatozoa.

Of note, subfertility appears to be a species-specific defect as no fertility problems have been reported in *Unga* deficient mice [71], and the only human phenotype related to mutations in *UNG* is immunodeficiency with hyper-IgM type 5 (HIGM5) (OMIM: 608106) [72]. Complementary expression data show that *mbd4* and the *tdg.2* paralog of *TDG* are both highly expressed in late stage spermatogenic cells, while *unga* expression dominates in oocytes (Figure S6). Functional distribution into various types of tissue mirrors observations in mice and humans, where UNG is essential for immunoglobulin diversification in B cells [72], whereas MBD4 and TDG prevent mutations at methylated CpG sites primarily in somatic tissues [73].

Zebrafish spermatogenesis has been thoroughly characterized in recent years, both at the level of chromosomal dynamics [74] and gene expression dynamics [52]. Furthermore, recently, the conserved fertilization complex that bridges sperm and egg has been elucidated in high detail [75]. Even given these insights, however, it is not clear why the absence of Unga in males would lead to the observed fertility defects. It is possible that BER has some functions during the remodeling of chromatin during spermatogenesis: in zebrafish, unlike in mammals, overall changes in the DNA methylation profile are relatively minor; however, a global compaction of the chromatin can be observed [76]. Based on our data, we also cannot exclude the possibility that Unga has some other, non-enzymatic functions that are not essential for spermatogenesis but affect the fertilization process itself. Another explanation for the subfertility observed in *unga* mutant males could be related to the elevated presence of uracil in the genomic DNA of the spermatozoa. Maternal components of the BER pathway present in the oocyte could trigger a fragmentation of the uracil-rich paternal genome. However, as subfertility has been observed in the in-cross of *unga* homozygotes (where the oocytes themselves would also lack Unga), even if this is the case, the activation of BER would be triggered by some alternative pathways (Note that Smug1, another UDG, appears to be absent from oocytes, based on the available datasets). Therefore, understanding the exact causes of subfertility in *unga* males will be an important future research program, especially as other fish species also show elevated expression levels of this gene both in their testes and their ovaries (Figure S8) [53].

While we currently cannot distinguish whether the increased apoptotic signal in DEB-treated *unga* mutants arises from elevated apoptosis or impaired clearance of apoptotic debris (efferocytosis), both outcomes are consistent with the observed phenotype. Clarifying this distinction will further illuminate the effects of genotoxic stress on Unga function. Importantly, these findings enhance our understanding of how DNA repair pathways respond to genotoxic events in zebrafish, particularly in rapidly dividing populations like the retinal progenitors of 2 dpf embryos. The requirement for Unga in these cells underscores its role in replication-associated uracil removal, with the observed apoptotic clusters likely reflecting accumulated uracil or abasic sites during DNA synthesis.

Finally, it is important to recognize the differences between our results and previous results, where the knockdown of *unga* with morpholino oligonucleotides led to embryo lethality [33]. While ultimately, only a detailed transcriptomic analysis will be able to reveal the differences between the knockdown and knockout embryos, we note that morpholino oligonucleotides have often been associated with numerous side effects, often with significant phenotypic consequences [39,40,77,78]. We also note that previously another frameshift *unga* allele (*unga^cu3^*) was already created and briefly mentioned in the literature but with no overall phenotype reported [21].

Overall, our study provides important insights into the function of *unga* in zebrafish genomic maintenance and reproductive biology and provides an overall characterization of the first bona fide BER mutant in zebrafish, to our knowledge. Our reanalysis of existing scRNAseq datasets also suggests a nuanced interplay between UDGs, hinting at the possible specialization of uracil-processing enzymes during spermatogenesis. The complementarity in expression and the distinct phenotypes upon *unga* loss support a model wherein uracil repair enzymes are tailored to specific germline lineages and developmental contexts.

## Data Availability

Datasets related to the GCF_049306965.1-RS_2025_04 zebrafish genome annotation are available through the NCBI website. Original data and all relevant R scripts used to analyze the data and to create the figures in the paper are openly available in the related GitHub repository: https://github.com/danio-elte/2025Kazzazy_unga_paper (accessed on 25 August 2025). Additionally, all raw datasets and scripts used to perform their analysis have also been deposited to Zenodo [79].

## Supplementary Materials

The following supporting information can be downloaded at: https://www.mdpi.com/article/10.3390/jdb13030032/s1, Figure S1: Loss and gain of *UNG* paralogs; Figure S2: Tissue-specific heatmaps showing the expression of *unga* and the top 50 co-expressed genes in the Daniocell dataset; Figure S3: Heatmaps specific for developmental stage (shown in hpf at the bottom) presenting the expression of *unga* and the top 50 co-expressed genes in the Daniocell dataset; Figure S4: Sanger sequencing a result of a homozygous *unga^elu24/elu24^* animal; Figure S5: Size comparison of 17 months old wild type and *unga^−/−^* mutant adult zebrafish; Figure S6: Expression of zebrafish genes coding UDG orthologs in the gonads; Figure S7: No excess cell death can be detected in the testes of *unga^−/−^* mutant adult males; Figure S8: Relative expression of *unga* orthologs (using log_2_ transformation) in the adult tissues and embryos of other fish species; Table S1: List of genes coexpressed with unga in the Daniocell dataset [x].

## Abbreviations

The following abbreviations are used in this manuscript:

AO	Acridine Orange
BER	Base Excision Repair
Blm	Bloom helicase
CASA	Computer-Assisted Semen Analysis
CDS	Coding sequence
CRISPR	Clustered Regularly Interspaced Short Palindromic Repeats
Cas9	CRISPR-associated protein 9
DSB	Double-stranded break
GO	Gene ontology
HE	Hematoxylin and eosin
HR	Homologous recombination
MMR	Mismatch repair
NER	Nucleotide excision repair
NHEJ	Non-homologous end-joining
NMD	Nonsense-mediated decay
PBS	Phosphate-buffered saline
PTU	1-phenyl 2-thiourea
UNG/UDG	Uracil-DNA glycosylase
ZGA	Zygotic genome activation

## References

[1] Hakem R. (2008). DNA-damage Repair; the Good, the Bad, and the Ugly. EMBO J..

[2] Cayuela M.L., Claes K.B.M., Ferreira M.G., Henriques C.M., Eeden F.V., Varga M., Vierstraete J., Mione M.C. (2019). The Zebrafish as an Emerging Model to Study DNA Damage in Aging, Cancer and Other Diseases. Front. Cell Dev. Biol..

[3] Shin U., Lee Y. (2023). Unraveling DNA Repair Processes In Vivo: Insights from Zebrafish Studies. Int. J. Mol. Sci..

[4] Dey A., Flajšhans M., Pšenička M., Gazo I. (2023). DNA Repair Genes Play a Variety of Roles in the Development of Fish Embryos. Front. Cell Dev. Biol..

[5] Lieschke G.J., Currie P.D. (2007). Animal Models of Human Disease: Zebrafish Swim into View. Nat. Rev. Genet..

[6] Kawakami K., Largaespada D.A., Ivics Z. (2017). Transposons As Tools for Functional Genomics in Vertebrate Models. Trends Genet..

[7] Varga M. (2018). The Doctor of Delayed Publications: The Remarkable Life of George Streisinger (1927–1984). Zebrafish.

[8] Liu K., Petree C., Requena T., Varshney P., Varshney G.K. (2019). Expanding the CRISPR Toolbox in Zebrafish for Studying Development and Disease. Front. Cell Dev. Biol..

[9] Uribe-Salazar J.M., Kaya G., Sekar A., Weyenberg K., Ingamells C., Dennis M.Y. (2022). Evaluation of CRISPR Gene-Editing Tools in Zebrafish. BMC Genom..

[10] Bedell V.M., Dubey P., Lee H.B., Bailey D.S., Anderson J.L., Jamieson-Lucy A., Xiao R., Leonard E.V., Falk M.J., Pack M.A. (2025). Zebrafishology, Study Design Guidelines for Rigorous and Reproducible Data Using Zebrafish. Commun. Biol..

[11] Wangler M.F., Yamamoto S., Chao H.-T., Posey J.E., Westerfield M., Postlethwait J.H., Hieter P., Boycott K.M., Campeau P.M., Members of the Undiagnosed Diseases Network (UDN) (2017). Model Organisms Facilitate Rare Disease Diagnosis and Therapeutic Research. Genetics.

[12] Varga M., Ralbovszki D., Balogh E., Hamar R., Keszthelyi M., Tory K. (2018). Zebrafish Models of Rare Hereditary Pediatric Diseases. Diseases.

[13] Patton E.E., Zon L.I., Langenau D.M. (2021). Zebrafish Disease Models in Drug Discovery: From Preclinical Modelling to Clinical Trials. Nat. Rev. Drug Discov..

[14] White R.M., Patton E.E. (2023). Adult Zebrafish as Advanced Models of Human Disease. Dis. Model. Mech..

[15] Botthof J.G., Bielczyk-Maczyńska E., Ferreira L., Cvejic A. (2017). Loss of the Homologous Recombination Gene Rad51 Leads to Fanconi Anemia-like Symptoms in Zebrafish. Proc. Natl. Acad. Sci. USA.

[16] Ramanagoudr-Bhojappa R., Carrington B., Ramaswami M., Bishop K., Robbins G.M., Jones M., Harper U., Frederickson S.C., Kimble D.C., Sood R. (2018). Multiplexed CRISPR/Cas9-Mediated Knockout of 19 Fanconi Anemia Pathway Genes in Zebrafish Revealed Their Roles in Growth, Sexual Development and Fertility. PLoS Genet..

[17] Rodríguez-Marí A., Wilson C., Titus T.A., Cañestro C., Bremiller R.A., Yan Y.-L., Nanda I., Johnston A., Kanki J.P., Gray E.M. (2011). Roles of Brca2 (Fancd1) in Oocyte Nuclear Architecture, Gametogenesis, Gonad Tumors, and Genome Stability in Zebrafish. PLoS Genet..

[18] Rodríguez-Marí A., Cañestro C., Bremiller R.A., Nguyen-Johnson A., Asakawa K., Kawakami K., Postlethwait J.H. (2010). Sex Reversal in Zebrafish Fancl Mutants Is Caused by Tp53-Mediated Germ Cell Apoptosis. PLoS Genet..

[19] Shive H.R., West R.R., Embree L.J., Azuma M., Sood R., Liu P., Hickstein D.D. (2010). Brca2 in Zebrafish Ovarian Development, Spermatogenesis, and Tumorigenesis. Proc. Natl. Acad. Sci. USA.

[20] Annus T., Müller D., Jezsó B., Ullaga G., Németh B., Harami G.M., Orbán L., Kovács M., Varga M. (2022). Bloom Syndrome Helicase Contributes to Germ Line Development and Longevity in Zebrafish. Cell Death Dis..

[21] Shin U., Nakhro K., Oh C.-K., Carrington B., Song H., Varshney G.K., Kim Y., Song H., Jeon S., Robbins G. (2021). Large-Scale Generation and Phenotypic Characterization of Zebrafish CRISPR Mutants of DNA Repair Genes. DNA Repair.

[22] Feitsma H., Kuiper R.V., Korving J., Nijman I.J., Cuppen E. (2008). Zebrafish with Mutations in Mismatch Repair Genes Develop Neurofibromas and Other Tumors. Cancer Res..

[23] Frederico L.A., Kunkel T.A., Shaw B.R. (1990). A Sensitive Genetic Assay for the Detection of Cytosine Deamination: Determination of Rate Constants and the Activation Energy. Biochemistry.

[24] Roberts J.Z., LaBonte M.J. (2023). The Importance of the Fifth Nucleotide in DNA: Uracil. Oligonucleotides—Overview and Applications.

[25] Lirussi L., Nilsen H.L. (2023). DNA Glycosylases Define the Outcome of Endogenous Base Modifications. Int. J. Mol. Sci..

[26] Otterlei M., Warbrick E., Nagelhus T.A., Haug T., Slupphaug G., Akbari M., Aas P.A., Steinsbekk K., Bakke O., Krokan H.E. (1999). Post-replicative Base Excision Repair in Replication Foci. EMBO J..

[27] Torseth K., Doseth B., Hagen L., Olaisen C., Liabakk N.-B., Græsmann H., Durandy A., Otterlei M., Krokan H.E., Kavli B. (2012). The UNG2 Arg88Cys Variant Abrogates RPA-Mediated Recruitment of UNG2 to Single-Stranded DNA. DNA Repair.

[28] Schormann N., Ricciardi R., Chattopadhyay D. (2014). Uracil-DNA Glycosylases—Structural and Functional Perspectives on an Essential Family of DNA Repair Enzymes. Protein Sci..

[29] Visnes T., Doseth B., Pettersen H.S., Hagen L., Sousa M.M.L., Akbari M., Otterlei M., Kavli B., Slupphaug G., Krokan H.E. (2009). Uracil in DNA and Its Processing by Different DNA Glycosylases. Philos. Trans. R. Soc. B Biol. Sci..

[30] Xue J.-H., Xu G.-F., Gu T.-P., Chen G.-D., Han B.-B., Xu Z.-M., Bjørås M., Krokan H.E., Xu G.-L., Du Y.-R. (2016). Uracil-DNA Glycosylase UNG Promotes Tet-Mediated DNA Demethylation. J. Biol. Chem..

[31] Bellacosa A., Drohat A.C. (2015). Role of Base Excision Repair in Maintaining the Genetic and Epigenetic Integrity of CpG Sites. DNA Repair.

[32] Thisse B., Thisse C. (2004). Fast Release Clones: A High Throughput Expression Analysis.

[33] Wu D., Chen L., Sun Q., Wu X., Jia S., Meng A. (2014). Uracil-DNA Glycosylase Is Involved in DNA Demethylation and Required for Embryonic Development in the Zebrafish Embryo. J. Biol. Chem..

[34] Bregenhorn S., Kallenberger L., Artola-Borán M., Peña-Diaz J., Jiricny J. (2016). Non-Canonical Uracil Processing in DNA Gives Rise to Double-Strand Breaks and Deletions: Relevance to Class Switch Recombination. Nucleic Acids Res..

[35] Chon J., Field M.S., Stover P.J. (2019). Deoxyuracil in DNA and Disease: Genomic Signal or Managed Situation?. DNA Repair.

[36] Békési A., Holub E., Pálinkás H.L., Vértessy B.G. (2021). Detection of Genomic Uracil Patterns. Int. J. Mol. Sci..

[37] Krokan H.E., Sætrom P., Aas P.A., Pettersen H.S., Kavli B., Slupphaug G. (2014). Error-Free versus Mutagenic Processing of Genomic Uracil—Relevance to Cancer. DNA Repair.

[38] Fortier S., Yang X., Wang Y., Bennett R.A.O., Strauss P.R. (2009). Base Excision Repair in Early Zebrafish Development: Evidence for DNA Polymerase Switching and Standby AP Endonuclease Activity. Biochemistry.

[39] Schulte-Merker S., Stainier D.Y.R. (2014). Out with the Old, in with the New: Reassessing Morpholino Knockdowns in Light of Genome Editing Technology. Development.

[40] Stainier D.Y.R., Raz E., Lawson N.D., Ekker S.C., Burdine R.D., Eisen J.S., Ingham P.W., Schulte-Merker S., Yelon D., Weinstein B.M. (2017). Guidelines for Morpholino Use in Zebrafish. PLoS Genet..

[41] Westerfield M. (2000). The Zebrafish Book.

[42] Aleström P., D’Angelo L., Midtlyng P.J., Schorderet D.F., Schulte-Merker S., Sohm F., Warner S. (2019). Zebrafish: Housing and Husbandry Recommendations. Lab. Anim..

[43] Kroll F., Powell G.T., Ghosh M., Gestri G., Antinucci P., Hearn T.J., Tunbak H., Lim S., Dennis H.W., Fernandez J.M. (2021). A Simple and Effective F0 Knockout Method for Rapid Screening of Behaviour and Other Complex Phenotypes. eLife.

[44] Meeker N.D., Hutchinson S.A., Ho L., Trede N.S. (2007). Method for Isolation of PCR-Ready Genomic DNA from Zebrafish Tissues. Biotechniques.

[45] Hu Y., Xie S., Yao J. (2016). Identification of Novel Reference Genes Suitable for qRT-PCR Normalization with Respect to the Zebrafish Developmental Stage. PLoS ONE.

[46] Bhat P., Cabrera-Quio L.E., Herzog V.A., Fasching N., Pauli A., Ameres S.L. (2023). SLAMseq Resolves the Kinetics of Maternal and Zygotic Gene Expression during Early Zebrafish Embryogenesis. Cell Rep..

[47] Posit Team (2025). RStudio: Integrated Development Environment for R. Posit Software.

[48] Sur A., Wang Y., Capar P., Margolin G., Prochaska M.K., Farrell J.A. (2023). Single-Cell Analysis of Shared Signatures and Transcriptional Diversity during Zebrafish Development. Dev. Cell.

[49] Satija R., Farrell J.A., Gennert D., Schier A.F., Regev A. (2015). Spatial Reconstruction of Single-Cell Gene Expression Data. Nat. Biotechnol..

[50] Yu G., Wang L.-G., Han Y., He Q.-Y. (2012). ClusterProfiler: An R Package for Comparing Biological Themes Among Gene Clusters. OMICS J. Integr. Biol..

[51] Liu Y., Kossack M.E., McFaul M.E., Christensen L.N., Siebert S., Wyatt S.R., Kamei C.N., Horst S., Arroyo N., Drummond I.A. (2022). Single-Cell Transcriptome Reveals Insights into the Development and Function of the Zebrafish Ovary. eLife.

[52] Sposato A.L., Hollins H.L., Llewellyn D.R., Weber J.M., Schrock M.N., Farrell J.A., Gagnon J.A. (2024). Germ Cell Progression through Zebrafish Spermatogenesis Declines with Age. Development.

[53] Pasquier J., Cabau C., Nguyen T., Jouanno E., Severac D., Braasch I., Journot L., Pontarotti P., Klopp C., Postlethwait J.H. (2016). Gene Evolution and Gene Expression after Whole Genome Duplication in Fish: The PhyloFish Database. BMC Genom..

[54] Letunic I., Bork P. (2024). Interactive Tree of Life (ITOL) v6: Recent Updates to the Phylogenetic Tree Display and Annotation Tool. Nucleic Acids Res..

[55] Wickham H. (2016). ggplot2: Elegant Graphics for Data Analysis.

[56] Kassambara A. (2023). ggpubr: “ggplot2” Based Publication Ready Plots. https://cran.r-project.org/web/packages/ggpubr/index.html.

[57] Schindelin J., Arganda-Carreras I., Frise E., Kaynig V., Longair M., Pietzsch T., Preibisch S., Rueden C., Saalfeld S., Schmid B. (2012). Fiji: An Open-Source Platform for Biological-Image Analysis. Nat. Methods.

[58] Sullivan-Brown J., Bisher M.E., Burdine R.D. (2011). Embedding, Serial Sectioning and Staining of Zebrafish Embryos Using JB-4 Resin. Nat. Protoc..

[59] Popovic N.T., Strunjak-Perovic I., Coz-Rakovac R., Barisic J., Jadan M., Berakovic A.P., Klobucar R.S. (2012). Tricaine Methane-sulfonate (MS-222) Application in Fish Anaesthesia. J. Appl. Ichthyol..

[60] Zanin M., Junior A.S.V., Acosta I.B., Gheller S.M.M., Zimermann E., Froes C.N., Gehrcke M.I., Corcini C.D. (2021). Tricaine Methanesulfonate (MS-222) on the Spermatic Quality of Zebrafish, *Danio rerio*. Aquaculture.

[61] Saad A., Billard R. (1987). Spermatozoa Production and Volume of Semen Collected after Hormonal Stimulation in the Carp, *Cyprinus Carpio*. Aquaculture.

[62] Kollár T., Kása E., Ferincz Á., Urbányi B., Csenki-Bakos Z., Horváth Á. (2018). Development of an in Vitro Toxicological Test System Based on Zebrafish (*Danio rerio*) Sperm Analysis. Environ. Sci. Pollut. Res..

[63] Elmi A., Casalini A., Bertocchi M., Emmanuele P., Aniballi C., Parmeggiani A., Govoni N., Ventrella D., Mordenti O., Bacci M.L. (2023). Comparative Evaluation of the Effects of Different Activating Media and Temperatures on European Eel (*Anguilla anguilla*) Sperm Motility Assessed by Computer Assisted Sperm Analysis. Res. Vet. Sci..

[64] Gentile L., Hausz B.L., Casalini A., Govoni N., Emmanuele P., Parmeggiani A., Ventrella D., Bacci M.L., Mordenti O., Elmi A. (2025). Milt Androgen Profile and Evaluation of Sperm Morpho-Functional Characteristics of Wild-Caught and Farmed European Eels (*Anguilla anguilla*). Fish Physiol. Biochem..

[65] Glasauer S.M.K., Neuhauss S.C.F. (2014). Whole-Genome Duplication in Teleost Fishes and Its Evolutionary Consequences. Mol. Genet. Genom..

[66] Qi M., Clark J., Moody E.R.R., Pisani D., Donoghue P.C.J. (2024). Molecular Dating of the Teleost Whole Genome Duplication (3R) Is Compatible With the Expectations of Delayed Rediploidization. Genome Biol. Evol..

[67] Otterlei M., Haug T., Nagelhus T.A., Slupphaug G., Lindmo T., Krokan H.E. (1998). Nuclear and Mitochondrial Splice Forms of Human Uracil-DNA Glycosylase Contain a Complex Nuclear Localisation Signal and a Strong Classical Mitochondrial Localisation Signal, Respectively. Nucleic Acids Res..

[68] da Silva Pescador G., Amaral D.B., Varberg J.M., Zhang Y., Hao Y., Florens L., Bazzini A.A. (2024). Protein Profiling of Zebrafish Embryos Unmasks Regulatory Layers during Early Embryogenesis. Cell Rep..

[69] Fishman L., Modak A., Nechooshtan G., Razin T., Erhard F., Regev A., Farrell J.A., Rabani M. (2024). Cell-Type-Specific MRNA Transcription and Degradation Kinetics in Zebrafish Embryogenesis from Metabolically Labeled Single-Cell RNA-Seq. Nat. Commun..

[70] Vértessy B.G., Tóth J. (2009). Keeping Uracil Out of DNA: Physiological Role, Structure and Catalytic Mechanism of dUTPases. Acc. Chem. Res..

[71] Nilsen H., Rosewell I., Robins P., Skjelbred C.F., Andersen S., Slupphaug G., Daly G., Krokan H.E., Lindahl T., Barnes D.E. (2000). Uracil-DNA Glycosylase (UNG)-Deficient Mice Reveal a Primary Role of the Enzyme during DNA Replication. Mol. Cell.

[72] Imai K., Slupphaug G., Lee W.-I., Revy P., Nonoyama S., Catalan N., Yel L., Forveille M., Kavli B., Krokan H.E. (2003). Human Uracil–DNA Glycosylase Deficiency Associated with Profoundly Impaired Immunoglobulin Class-Switch Recombination. Nat. Immunol..

[73] Cortázar D., Kunz C., Selfridge J., Lettieri T., Saito Y., MacDougall E., Wirz A., Schuermann D., Jacobs A.L., Siegrist F. (2011). Embryonic Lethal Phenotype Reveals a Function of TDG in Maintaining Epigenetic Stability. Nature.

[74] Imai Y., Olaya I., Sakai N., Burgess S.M. (2021). Meiotic Chromosome Dynamics in Zebrafish. Front. Cell Dev. Biol..

[75] Deneke V.E., Blaha A., Lu Y., Suwita J.P., Draper J.M., Phan C.S., Panser K., Schleiffer A., Jacob L., Humer T. (2024). A Conserved Fertilization Complex Bridges Sperm and Egg in Vertebrates. Cell.

[76] Ruíz A.M.B., Geng F.-S., Pujol G., Sanabria E., Brethouwer T., Almuedo-Castillo M., Ruiz-Herrera A., Tena J.J., Bogdanovic O. (2025). A Single-Cell Multiomics Roadmap of Zebrafish Spermatogenesis Reveals Regulatory Principles of Male Germline Formation. bioRxiv.

[77] Gentsch G.E., Spruce T., Monteiro R.S., Owens N.D.L., Martin S.R., Smith J.C. (2018). Innate Immune Response and Off-Target Mis-Splicing Are Common Morpholino-Induced Side Effects in Xenopus. Dev. Cell.

[78] Lai J.K.H., Gagalova K.K., Kuenne C., El-Brolosy M.A., Stainier D.Y.R. (2019). Induction of Interferon-Stimulated Genes and Cellular Stress Pathways by Morpholinos in Zebrafish. Dev. Biol..

[79] Kazzazy L., Huba F., Hausz B.L., Mező D., Perey-Simon V., Jezsó B., Seddik A., Marinović Z., Tóth J., Békési A. (2025). Raw Datasets and Related Scripts Used for the Descriptive Analysis of Zebrafish Unga Mutants. Zenodo. https://zenodo.org/records/16506721.

