# Supplementary material for: Zebrafish Unga Is Required for Genomic Maintenance upon Genotoxic Stress and Male Fertility
Source: J Dev Biol. 2025 Sep 2;13(3):32. doi: 10.3390/jdb13030032 (PMC12452583; doi:10.3390/jdb13030032)
Supplement: Supplementary file 1 [file jdb-13-00032-s001.zip › jdb-3708188-supplementary.pdf]

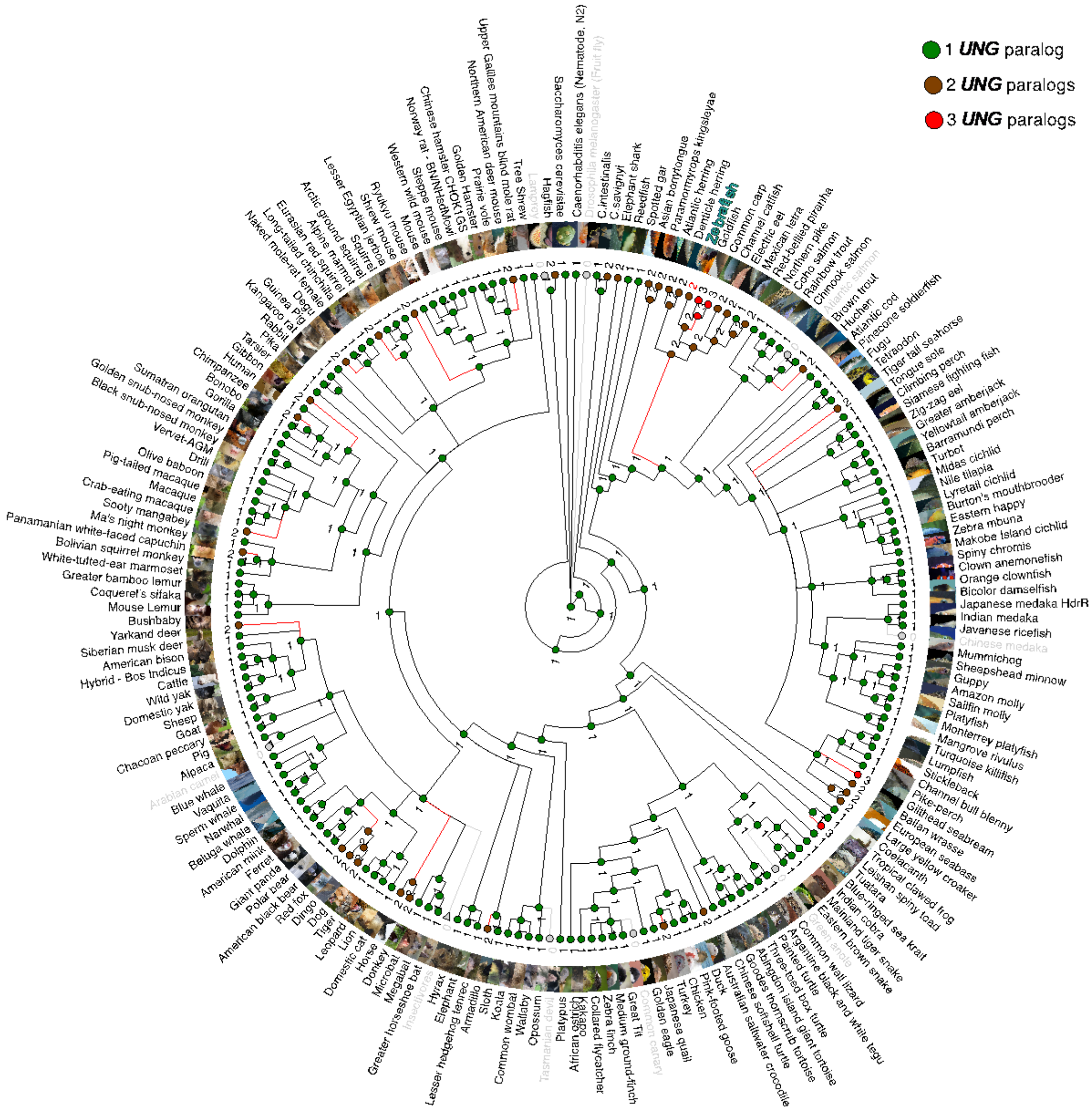

Figure S1: Phylogenetic analysis of *UNG* orthologs in vertebrates. The phylogenetic tree displays the number of *UNG* paralogs present in the genome of extant vertebrate species and their ancestors. This tree confirms that the presence of the two paralogs (*unga* and *ungb*) in the zebrafish genome is unrelated to the teleost-specific genome duplication (3R) event, and it is specific to the Otocephala clade. (Figure generated and downloaded from Ensembl.)

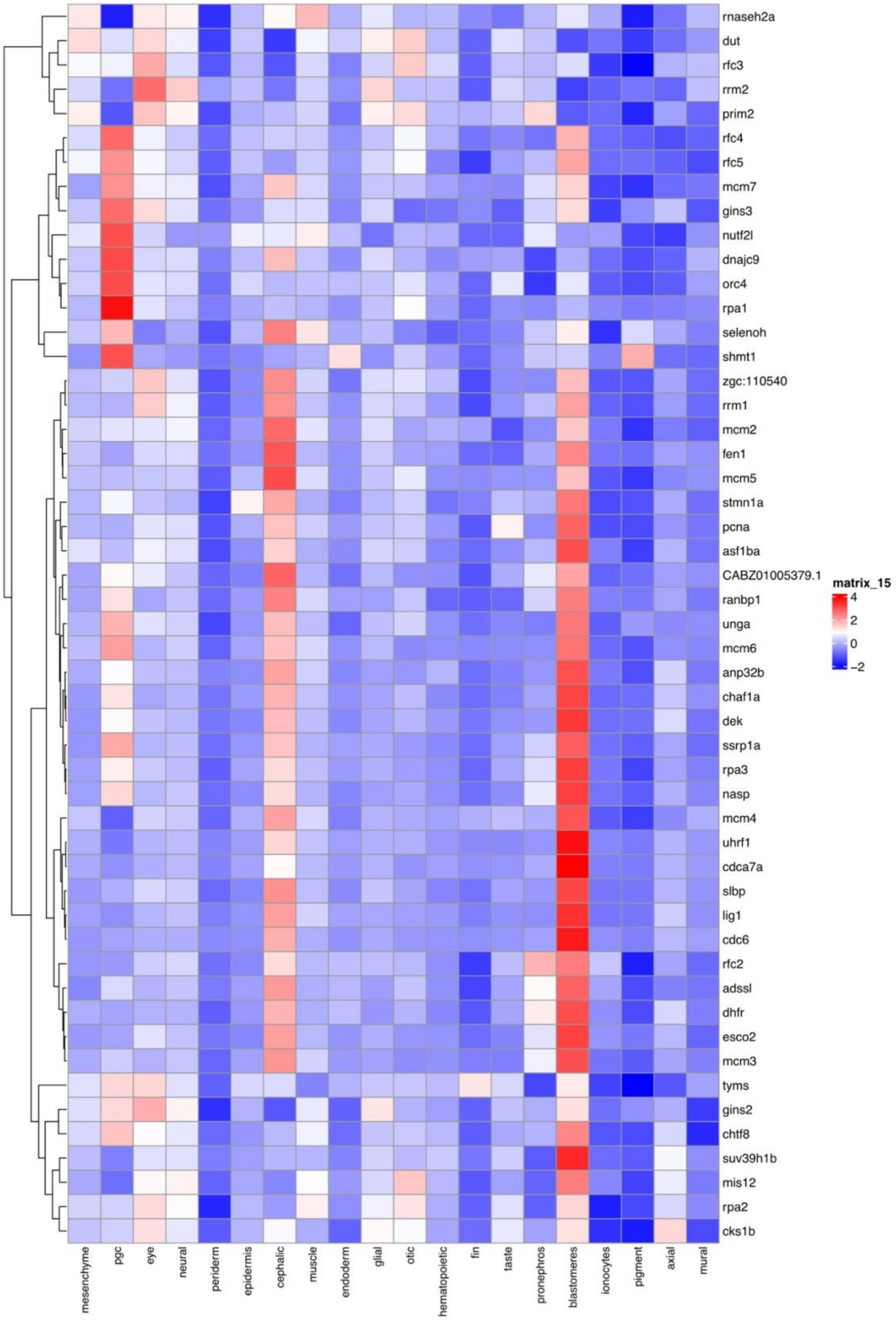

Figure S2: Tissue-specific heatmaps showing the expression of *unga* and the top 50 co-expressed genes in the Daniocell dataset [48].

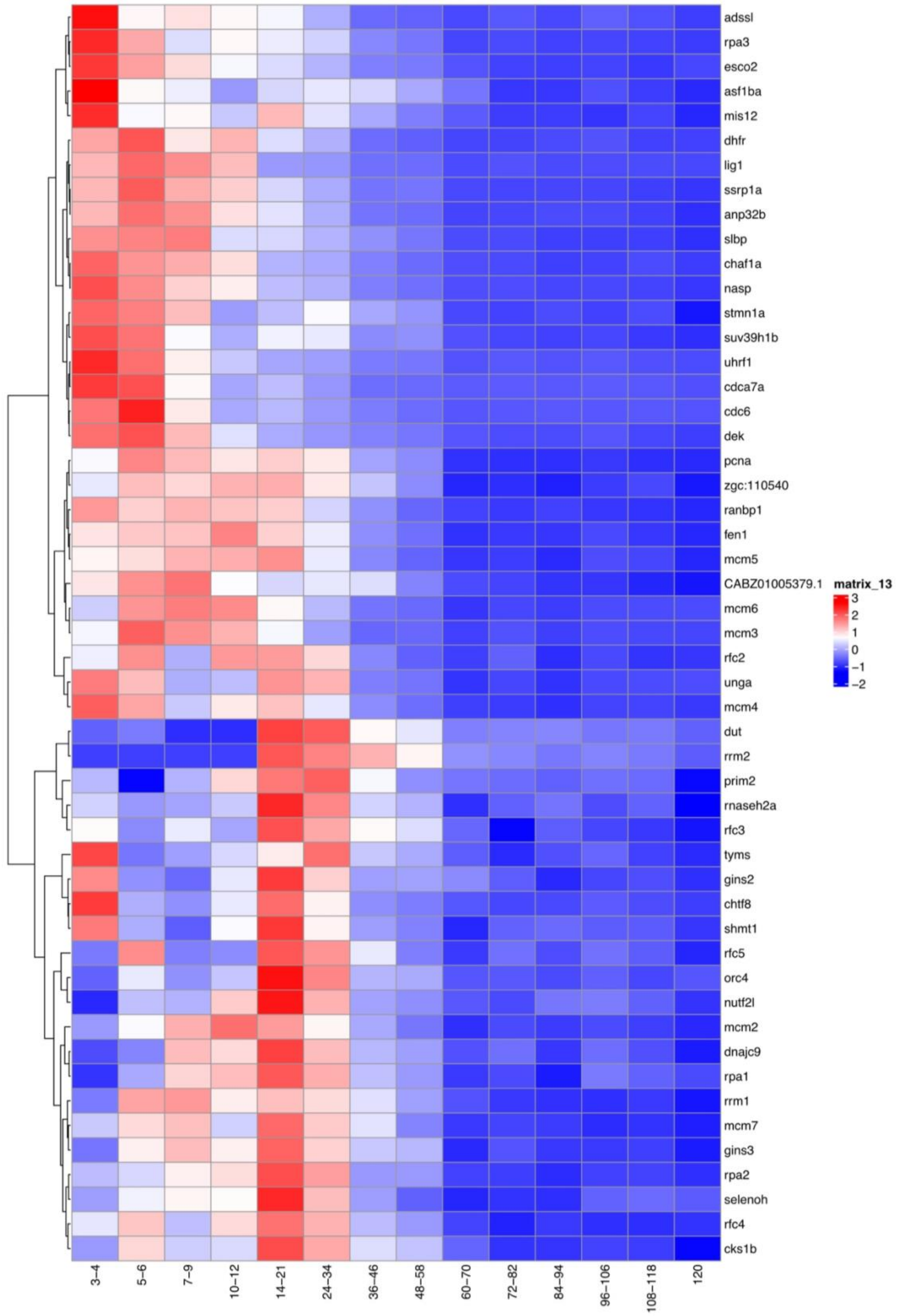

Figure S3: Heatmaps specific for developmental stage (shown in hpf at the bottom) presenting the expression of *unga* and the top 50 co-expressed genes in the Daniocell dataset [48].

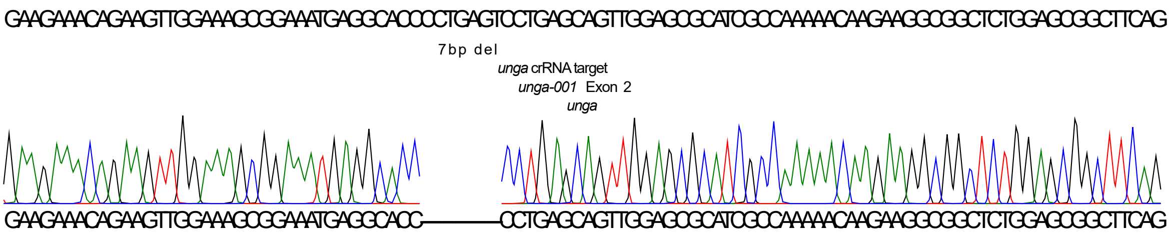

Figure S4: Sanger sequencing of a homozygous *unga*$^{elu24/elu24}$ zebrafish confirms the presence of a 7 bp long deletion at the targeting site.

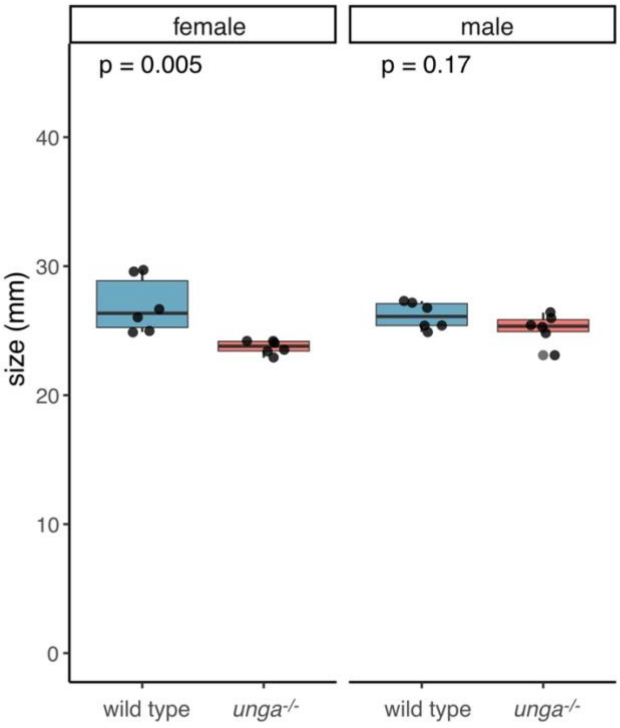

Figure S5: Size comparison of 17 months old wild type and *unga*$^{-/-}$ mutant adult zebrafish. Our results suggest a small, but significant size difference between age-matched control and mutant females, while no difference was detected in the size of the males. (The Mann-Whitney test was used to calculate p-values.)

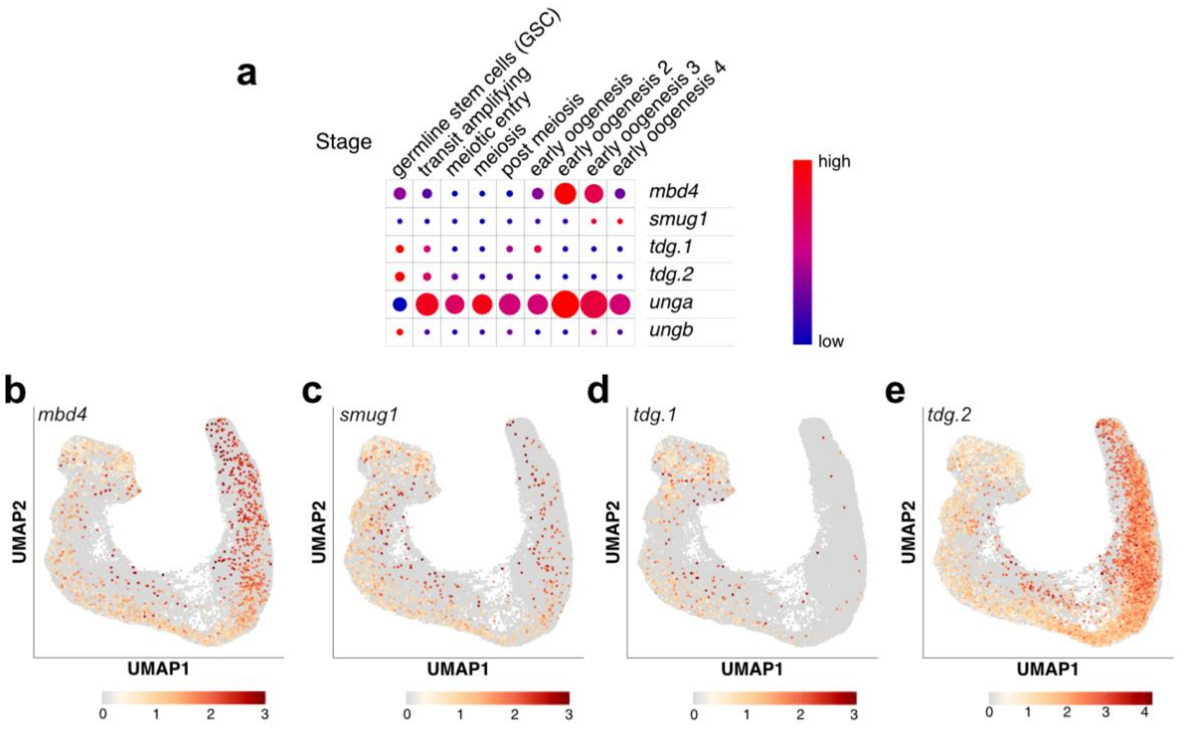

Figure S6: Expression of zebrafish genes coding UDG orthologs in the gonads. (**a**) Relative expression of *mbd4, smug1, tdg.1, tdg.2, unga* and *ungb* in adult ovary germ cells. (Based on data from: [51]) (**b**) Relative expression of *mbd4, smug1, tdg.1, tdg.2* in adult testicular germ cells. (Based on data from [52])

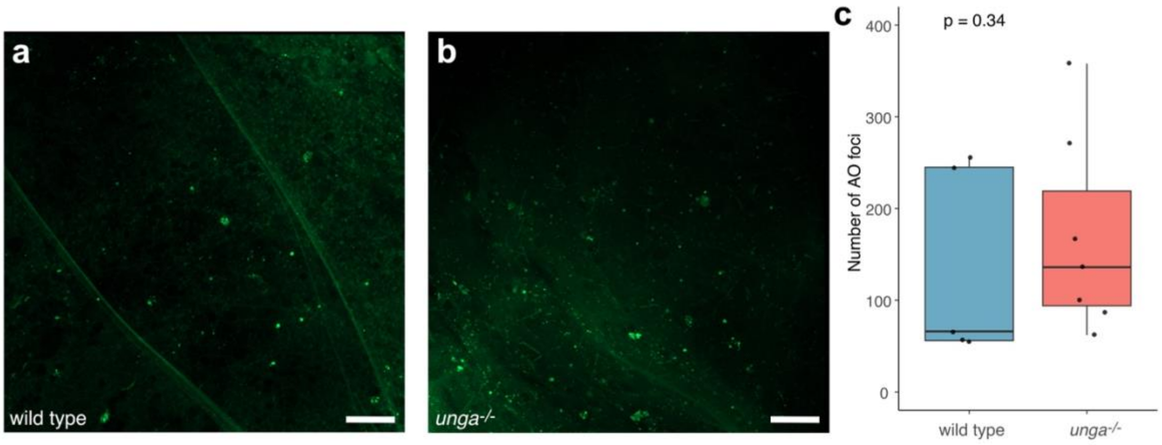

Figure S7: No excess cell death can be detected in the testes of *unga*$^{-/-}$ mutant adult males. (A,B) Acridin orange (AO) staining of control (A) and *unga*$^{-/-}$ mutant zebrafish testes. (C) Quantification of AO-positive foci in the testes of wild-type and *unga*$^{-/-}$ mutant zebrafish. (The Mann-Whitney test was used to calculate p-values.)

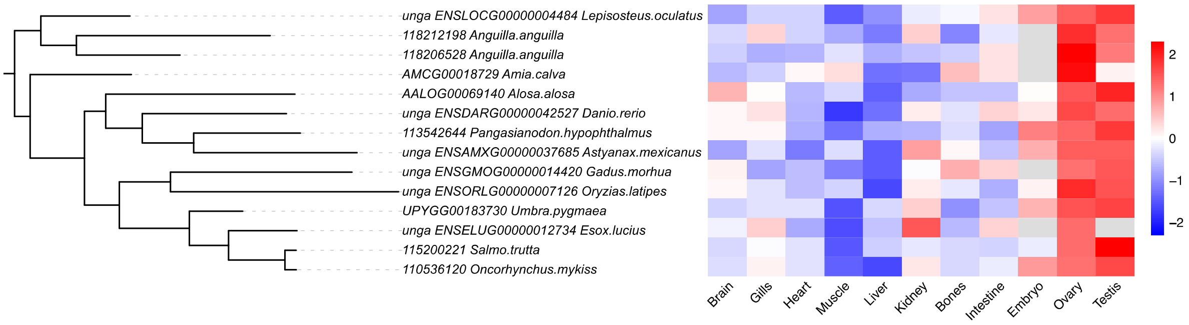

Figure S8: Relative expression of *unga* orthologs (using $\log_2$ transformation) in the adult tissues and embryos of other fish species. Grey squares indicate missing data. (Based on data from the PhyloFish database [53])

Table S1: List of genes coexpressed with *unga* in the Daniocell dataset [x].

gene	correlation
pcna	0.5118549
fen1	0.48091121
rpa2	0.47457049
zgc:110540	0.46803625
rpa3	0.46551068
rrm1	0.46146142
dut	0.45949118
esco2	0.45448482
chaf1a	0.45326702
nasp	0.45234997
lig1	0.45030276
mcm7	0.44895023
dhfr	0.44004717
slbp	0.43844292
CABZ01005379.1	0.43638198
orc4	0.43510061
mcm4	0.42960955
rfc4	0.4285117
rnaseh2a	0.42446764
stmn1a	0.4239482
mcm5	0.42154532
mcm6	0.41674569
rrm2	0.41248291
asf1ba	0.41027704
dnajc9	0.40809964
rfc3	0.40734743
mcm2	0.40545298
cdc6	0.40380024
rfc5	0.40362421
selenoh	0.40305919
rfc2	0.39987136
gins2	0.39867379
mcm3	0.39778118
prim2	0.39648489
nutf2l	0.39391283
suv39h1b	0.39215761
ssrp1a	0.39182647
uhrf1	0.38979093
dek	0.38953166
ranbp1	0.38590715

gene	correlation
tyms	0.3854612
rpa1	0.38533866
chtf8	0.38499418
shmt1	0.38378455
anp32b	0.38292486
cdca7a	0.38150748
cks1b	0.3815007
adssl	0.37943869
mis12	0.37921816
gins3	0.37900137
hells	0.37715728
mibp	0.37657513
msh6	0.37594366
tuba8l4	0.3755354
cdca7b	0.37543661
banf1	0.37491869
pola2	0.37447269
gins1	0.36936951
ccna2	0.36885914
tk1	0.36843706
si:ch211-156b7.4	0.36582548
XLOC-042899	0.36513862
hirip3	0.36405464
lbr	0.36247912
cdca5	0.36122591
ube2t	0.36042706
atad5a	0.35877412
si:dkey-6i22.5	0.35800413
sumo3b	0.35793318
haus4	0.35570753
msh2	0.35518082
ran	0.35511997
rnaseh2b	0.35421585
dnmt1	0.35355908
ppm1g	0.35277841
btg3	0.35260512
krcp	0.35244156
fbxo5	0.35199044
ccne2	0.35043913
LOC100330864	0.34937235

**gene**	**correlation**
gmnn	0.3477417
prim1	0.34691166
nup37	0.34566968
smc2	0.34468059
nsd2	0.3441867
cnbpa	0.3438457
snu13b	0.34365319
orc6	0.34003096
pola1	0.33858769
hpf1	0.33852041
hmgb2a	0.33789129
ccnd1	0.33755643
supt16h	0.3375056
usp1	0.33574869
ptgr1	0.33502389
nop58	0.33277097
dkc1	0.33195185
snrpd1	0.33077823
ncapg	0.32980583
cbx3a	0.3297842
mki67	0.32954525
mcmbp	0.32846858
cenpx	0.32798843
parp1	0.32719428
pa2g4a	0.32668939
cdk2	0.32445771
npm1a	0.32352607
rbbp4	0.32308072
dtymk	0.3230278
pa2g4b	0.32282908
chtf18	0.32183485
si:dkey-185e18.7	0.32177853
cad	0.32165271
hmgb2b	0.32055228
ivns1abpb	0.32045767
zgc:110216	0.31950034

**gene**	**correlation**
paics	0.31931477
asf1bb	0.31865795
tipin	0.31760892
vrk1	0.31686509
snrpb	0.31684816
kpnb3	0.31589008
dnph1	0.31535629
nap1l1	0.3144454
fbl	0.31274946
setb	0.31209669
si:dkey-261m9.17	0.31177595
ptges3b	0.31073836
nhp2	0.31028376
her2	0.31003083
smc4	0.30953784
ncapd2	0.30746914
nop56	0.30730082
hat1	0.30648077
siva1	0.30635836
polr2h	0.3061066
umps	0.30564245
rad51	0.30538005
nop10	0.3047932
parp2	0.30478427
CU929070.1	0.30468809
snrpf	0.30400914
pold3	0.30350408
abce1	0.3033637
rbb4l	0.30309697
aurkb	0.30306155
mad2l1	0.30271851
pole4	0.30256155
ctps1a	0.30155845
hmga1a	0.30105035
h2afx	0.30055051
pole3	0.30051071
seta	0.30025868
